# Supplementary figures and images for: Paired Feed-Forward Excitation With Delayed Inhibition Allows High Frequency Computations Across Brain Regions
Source: Front Neural Circuits. 2022 Feb 3;15:803065. doi: 10.3389/fncir.2021.803065 (PMC8862685; doi:10.3389/fncir.2021.803065)

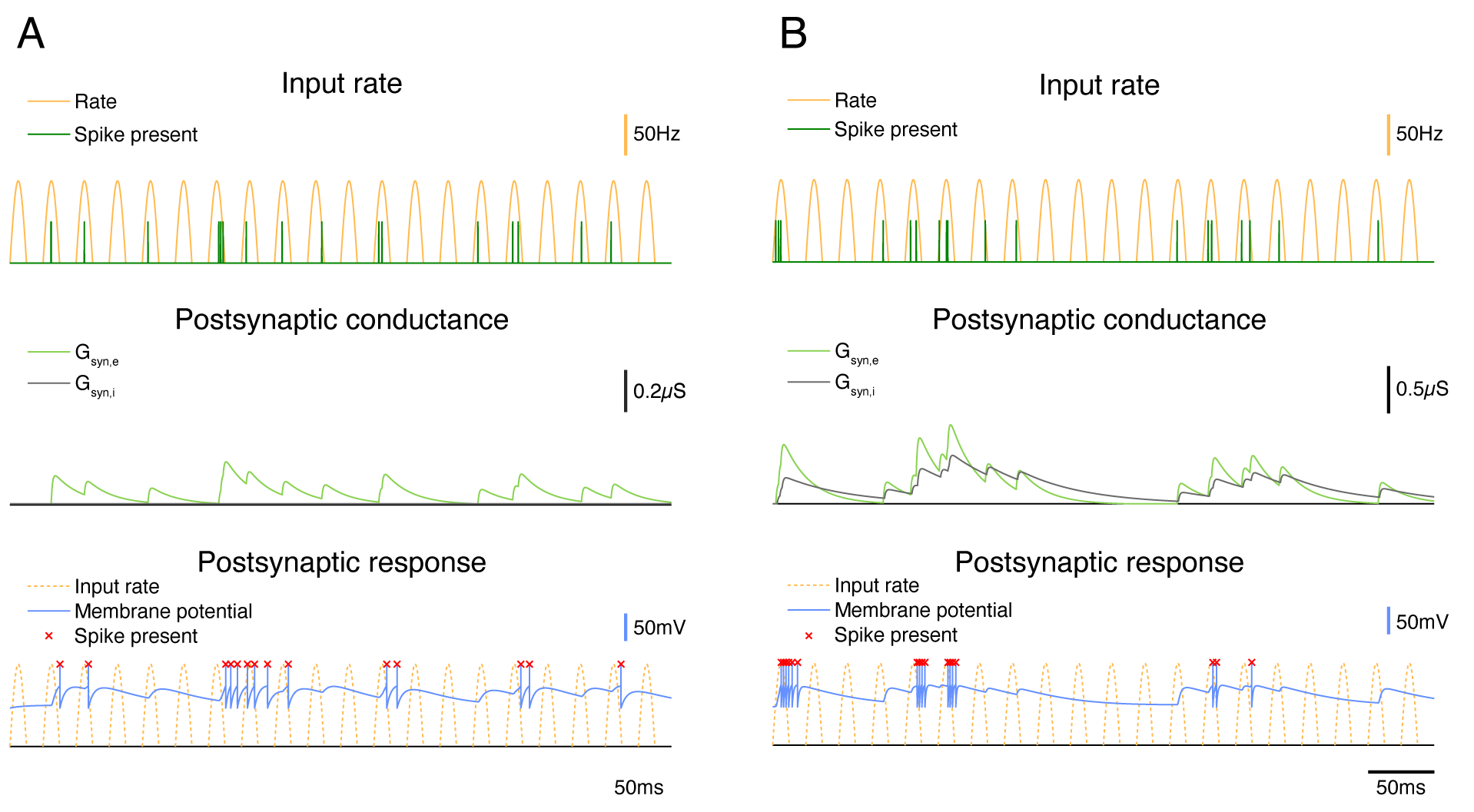

Supplement: Supplementary Figure 1 — High frequency transmission differs between excitation-only circuits and circuits exhibiting paired feed-forward excitation and delayed inhibition with long time constants. (A) 0.5 s simulation of a feed-forward E circuit receiving a single rectified sinusoidal Poisson input (yellow) with a peak rate (PR) of 100 Hz and an input modulation frequency (F) of 40 Hz, as in Figure 1A. (B) Same, but for a paired feed-forward E/I model circuit with τfall, i = 50 ms instead of the baseline 20 ms shown in Figure 1A. Spiking activity in the postsynaptic neuron with τfall, i = 50 ms is more prolonged than in the 20 ms case in Figure 1A, but the firing is still truncated by the inhibition, as compared to the no-inhibition case in panel (A). [file Image_1.tif]

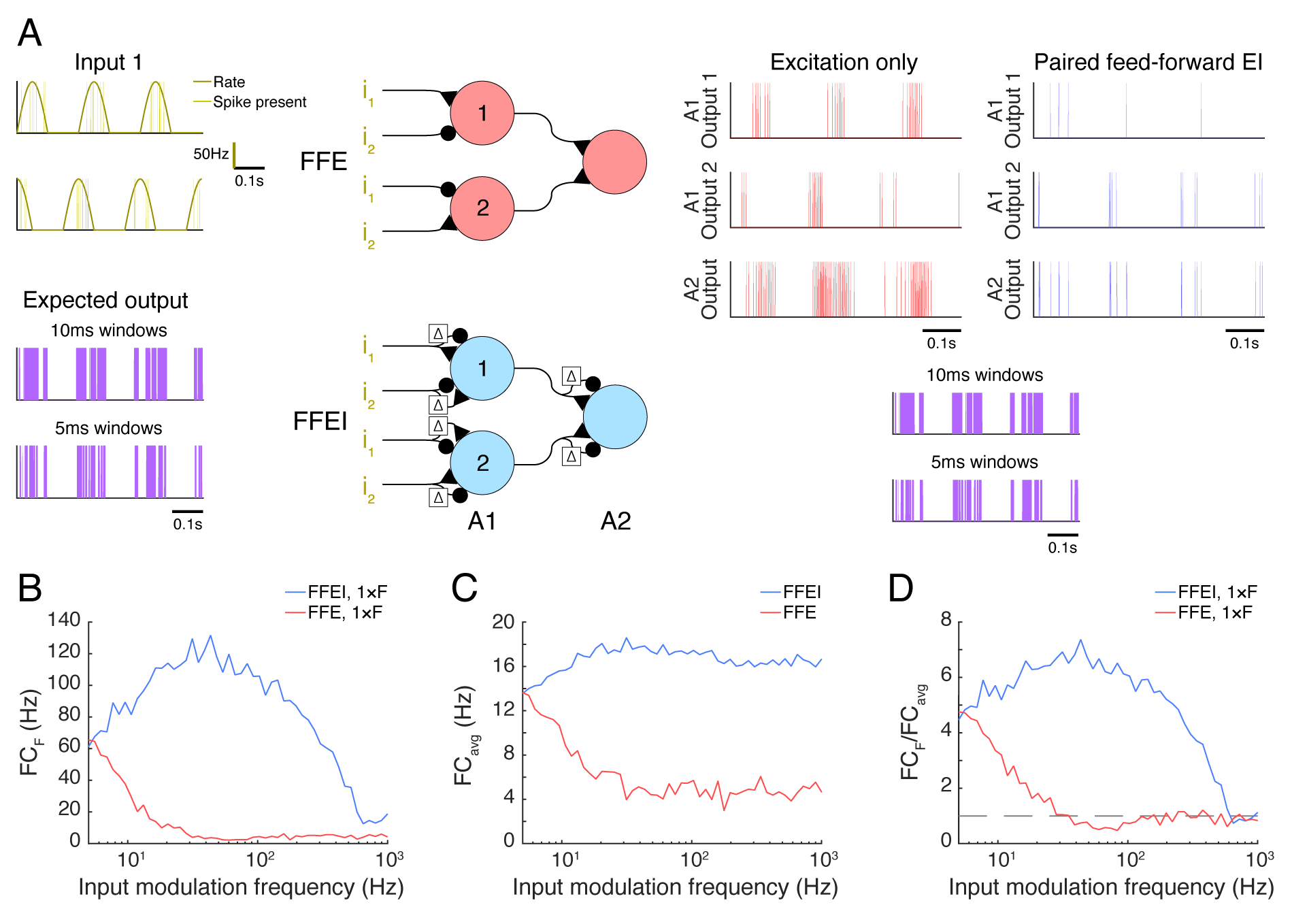

Supplement: Supplementary Figure 2 — Paired feed-forward E/I in an XOR circuit, with π/2 phase-shifted inputs. (A) A simulation of each XOR model circuit described in Figure 6A is shown with inputs modeled as in Figure 6A, but with a π/2 phase shift instead of a π phase shift, which should result in output at the stimulus frequency F (instead of 2 × F when the phase shift is π). (B) For a range of input modulation frequencies F, the Fourier coefficient of the A2 output at F (FCF) was averaged over 10 trials for the two XOR circuit models shown in (A). (C) For a range of input modulation frequencies F, the mean power of the A2 output over all frequencies (FCavg). (D) For a range of input modulation frequencies F, the normalized Fourier coefficient of the A2 output is shown. Dashed line indicates FCF/FCavg = 1. [file Image_2.tif]

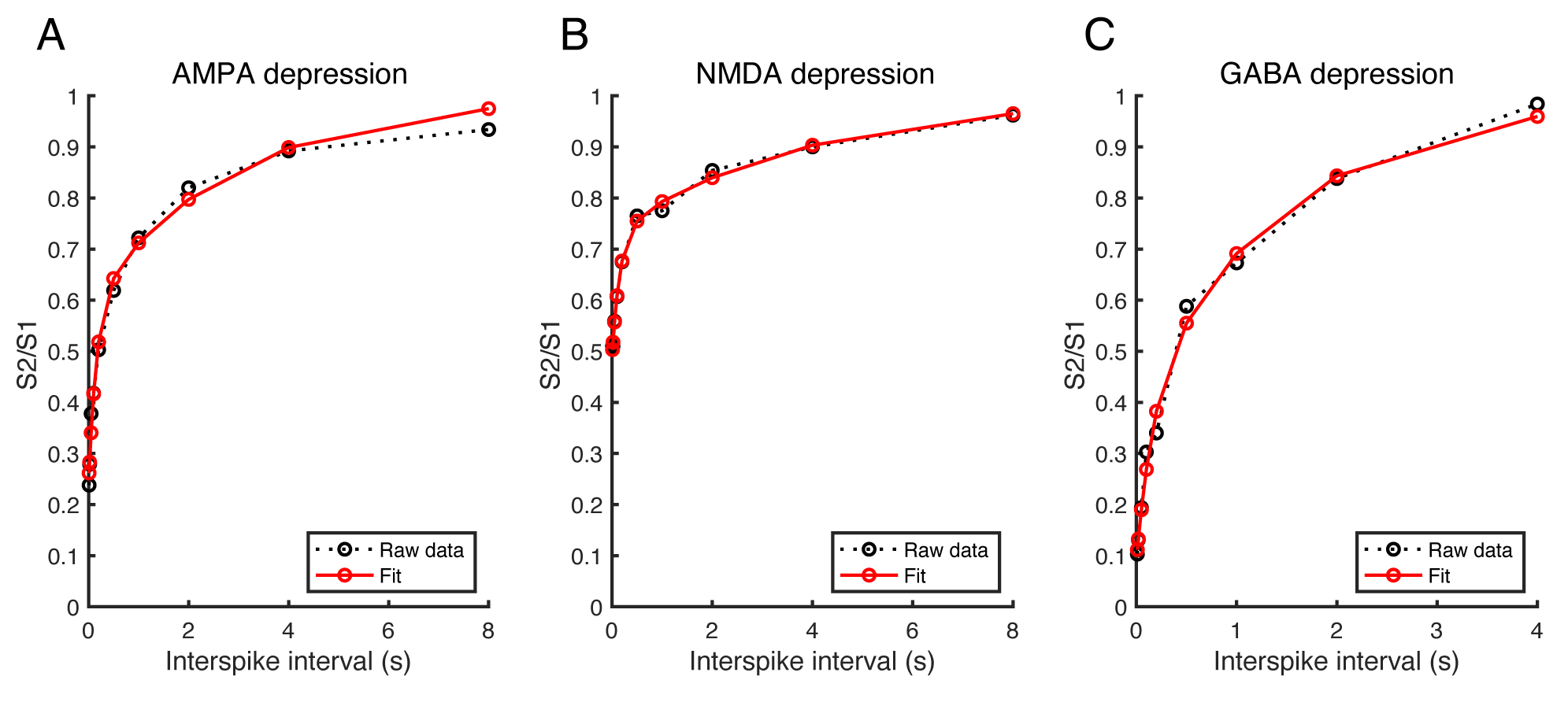

Supplement: Supplementary Figure 3 — Fitting depression at the retinogeniculate synapse. (A–C) The paired-pulse ratio for the AMPAR (A), NMDAR (B), and locked GABAR (C) components of the synaptic current was plotted over a range of interspike intervals. For each channel, raw data (black) obtained from voltage clamp experiments (Chen et al., 2002; Blitz and Regehr, 2005) were fitted to a depression model (see “Materials and Methods” section). Paired-pulse experiments were then simulated using the fitted depression model (red). [file Image_3.tif]
